# The Cold Hand Sign in Multiple System Atrophy: Frequency-Associated Factors and Its Impact on Survival

**DOI:** 10.3389/fnagi.2021.767211

**Published:** 2021-12-20

**Authors:** Bei Cao, Yan Liang, Ling-Yu Zhang, Yan-Bing Hou, Ru-Wei Ou, Qian-qian Wei, Huifang Shang

**Affiliations:** Laboratory of Neurodegenerative Disorders, Department of Neurology, Rare Diseases Center, National Clinical Research Center for Geriatrics, West China Hospital, Sichuan University, Chengdu, China

**Keywords:** multiple system atrophy (MSA), survival, cold hand sign, autonomic symptoms, non-motor symptoms (NMS)

## Abstract

**Background:** Few studies have focused on the cold hand sign (CHS), a red flag symptom, in multiple system atrophy (MSA).

**Objective:** This study aimed to investigate the frequency and correlative factors of CHS in patients with MSA and the impact of its early occurrence on the survival of these patients.

**Methods:** A total of 483 patients with MSA were enrolled in this study, and the motor and non-motor symptoms between patients with MSA with and without CHS were compared. Moreover, patients with disease duration ≤ 3 years at baseline were followed, and the association between CHS and survival of patients with MSA was examined.

**Results:** The frequencies of CHS in patients with MSA were 20, 15.4, and 25.3% in MSA, MSA-parkinsonian subtype (MSA-P), and MSA-cerebellar subtype (MSA-C), respectively. Higher Unified Multiple System Atrophy Rating Scale (UMSARS) scores and higher Non-Motor Symptom Scale (NMSS) scores at baseline were associated with CHS in MSA. CHS was associated with shorter survival after adjusting for baseline diagnosis subtype, age at onset, sex, orthostatic hypotension, disease duration, autonomic onset, UMSARS total score, and NMSS score (*p* = 0.001; HR = 3.701; 95% CI = 1.765–7.760).

**Conclusion:** CHS is not rare in patients with MSA. Greater disease severity and more severe non-motor symptoms were associated with CHS in patients with MSA. Patients with early occurrence of CHS had a poor prognosis.

## Introduction

Multiple system atrophy (MSA) is a fatal α-synucleinopathy characterized by a variable combination of autonomic dysfunction, parkinsonism, cerebellar ataxia, and pyramidal symptoms (Fanciulli and Wenning, 2015). Autonomic dysfunction is necessary for the diagnosis of MSA based on clinical diagnostic criteria (Gilman et al., 2008). Several longitudinal studies with large sample size and two autopsy-confirmed case studies have shown that severe dysautonomia and the early occurrence of autonomic failure predicted the poor prognosis of MSA (Coon et al., 2015; Low et al., 2015; Cao et al., 2018). Among the autonomic dysfunction symptoms, orthostatic hypotension (OH), urinary symptoms, and sexual dysfunction have attracted more attention because they are more common in patients (Fanciulli and Wenning, 2015).

However, the vasomotor system is also involved in the autonomic dysfunction in MSA (Klein et al., 1997; Yamanaka et al., 2007; Pietzarka et al., 2010; Augustis et al., 2017), such as the cold hand sign (CHS), which indicates that patients with MSA often have cold, dusky, and violaceous hands with poor circulatory return upon application of pressure. Previous studies have shown significantly different skin temperature, skin blood flow, and its response to cooling before and after cooling, as well as the kinetics of natural rewarming, compared with healthy subjects or patients with Parkinson’s disease (PD) (Klein et al., 1997; Pietzarka et al., 2010; Augustis et al., 2017). Meanwhile, a study on patients with MSA assessing skin vasomotor function in response to local heating revealed that MSA had reduced the amplitude of skin blood flow to heating compared with healthy patients (Yamanaka et al., 2007).

Klein et al. (1997) found that CHS was distinctive and common in MSA but rare in PD. In 2008, the European MSA Study Group (EMSA-SG) listed the “red flags,” which act as supportive criteria for the diagnosis of probable MSA, which included CHS (Kollensperger et al., 2008). However, to date, few small samples and cross-sectional studies (Kollensperger et al., 2008; Pietzarka et al., 2010; Asahina et al., 2013; Tandon and Pradhan, 2015) have investigated CHS in MSA. For example, a retrospective analysis of 57 patients with MSA-parkinsonian subtype (MSA-P) revealed that 39.3% of the patients with MSA-P had complained of CHS (Kollensperger et al., 2008), whereas another cross-sectional study, which enrolled 37 patients with MSA-cerebellar subtype (MSA-C) and 16 patients with MSA-P, found that 20% of patients with MSA had CHS (Tandon and Pradhan, 2015). In addition, these previous studies did not explore the correlative factors of its occurrence in MSA, such as subtype, disease severity, and other motor and non-motor symptoms. In addition, the role of early events of CHS in MSA in prognosticating survival is also largely unknown. Therefore, it is noteworthy to comprehensively investigate the frequency and correlative factors of CHS in MSA and explore the association between early CHS and the survival of patients with MSA.

## Materials and Methods

### Subjects

We enrolled patients who were diagnosed with “probable” MSA according to the established criteria (Gilman et al., 2008) at the Department of Neurology, West China Hospital, Sichuan University (a leading medical center in Southwest China) between January 2013 and November 2019. Informed written consent was obtained from all participants; then, approval was obtained from the Ethics Committee of West China Hospital of Sichuan University. Patients who met the “possible” diagnosis of MSA or who had incomplete data were excluded from this study. All patients were screened for spinocerebellar ataxia (*SCA*) genes, including *SCA1, SCA2, SCA3, SCA6*, and *SCA7*, to exclude the common forms of SCA. They were also subjected to brain MRI scans to exclude other structural neurological disorders. The patients were categorized as the MSA-C subtype when cerebellar ataxia symptoms and signs were observed predominately and categorized as the MSA-P subtype when parkinsonism symptoms and signs were observed predominately.

At baseline, all patients were evaluated *via* face-to-face interviews. Two neurologists independently examined each patient. Clinical information on sex, age at onset, motor symptoms (parkinsonism and cerebellar ataxia), autonomic symptoms, and neurological physical examination scores were recorded. Symptom onset was defined as the initial presentation of any motor symptoms (i.e., parkinsonism or cerebellar ataxia) or selected autonomic features, including OH or neurogenic bladder disturbances (Gilman et al., 2008). CHS refers to the new development of coldness and color change (purple/blue) of extremities, with blanching on pressure and poor circulatory return, as proposed by the EMSA-SG (Kollensperger et al., 2008), which was diagnosed at the baseline enrollment. Disease duration was defined as the time from the date of disease onset to the evaluation date. Rapid-eye-movement sleep behavior disorder (RBD) was diagnosed according to the International Classification of Sleep Disorders (Sateia, 2014). All participants underwent blood pressure (BP) testing to evaluate whether they had OH at the baseline visit. BP measurements were taken at intervals of 1, 3, 5, and 10 min in the upright position and were compared with the measurements in the supine position (baseline) (Gilman et al., 2008). Disease severity was rated using part I (activities of daily living, ADL), part II (motor examination), part III (autonomic examination), and part IV (global disability) of the Unified Multiple System Atrophy Rating Scale (UMSARS) (Wenning et al., 2004). The total UMSARS score is the sum of parts I and II. Other clinical assessments included the Montreal Cognitive Assessment (MoCA) (Nasreddine et al., 2005), Non-Motor Symptoms Scale (NMSS) (Chaudhuri et al., 2006), Hamilton Depression Rating Scale-24 items (HDRS) (Moberg et al., 2001), and Hamilton Anxiety Rating Scale (HARS) (Hamilton, 1959).

**FIGURE 1:**
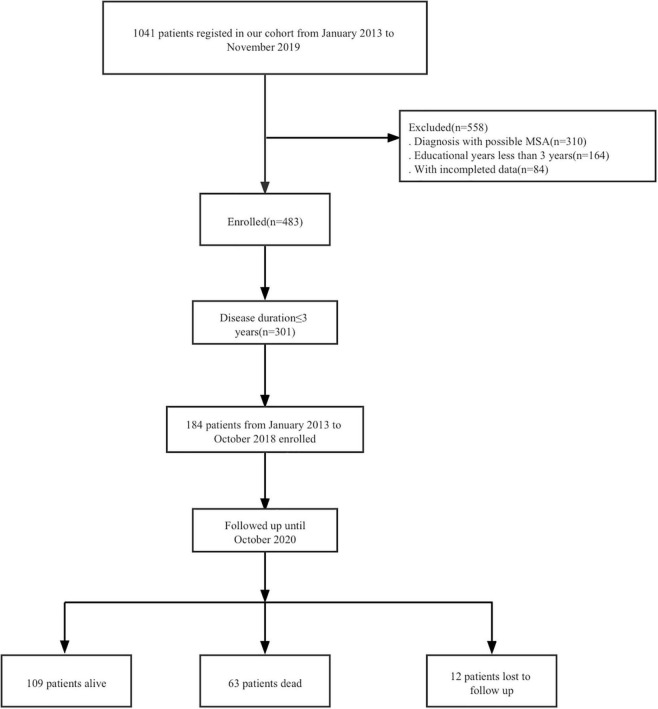
Study flow diagram.

To explore the impact of early occurrence of CHS in MSA, we followed up the patients with disease duration ≤ 3 years at baseline who were enrolled between January 2013 and October 2018 using telephone or face-to-face interviews in the 1-year interval until October 2020 with a median follow-up period of > 4 years (Figure 1). Loss to follow-up was due to a change in the telephone numbers or refusal to participate in more than three follow-up evaluations.

### Statistical Analysis

All continuous variables are expressed as the mean and standard deviation. The Kolmogorov–Smirnov test was used to assess normal distribution. Analysis of variance was performed to compare continuous variables in a normal distribution, whereas the Mann–Whitney *U*-test was performed to compare non-normally distributed continuous data between patients with and without CHS. After comparison, the significant variables were included as independent variables in the logistic regression analysis to investigate the potential factors related to CHS. The Kaplan–Meier curves were used to graphically analyze the overall death (or related death), and the log-rank test was performed to compare survival between patients with and without CHS. Univariate and multivariate Cox regression analyses were performed to explore the impact of CHS on survival. A list of potential risk factors for mortality was included in the univariate model, including the diagnosis subtype, age, sex, OH, disease duration, autonomic onset, total UMSARS score, and NMSS score. In the final multivariate models, variables related to survival (*p* < 0.1 in the univariate model) were included. Statistical significance was set at a two-tailed *p*-value < 0.05. Statistical analysis was performed using SPSS version 25.0 (SPSS, Chicago, IL, United States). Bonferroni correction for multiple comparisons was performed as appropriate.

## Results

### Clinical Features

Demographic and clinical data of patients with MSA, MSA-C, and MSA-P are shown in Table 1. In total, 483 patients with MSA were recruited in the current study, including 229 patients with MSA-P and 254 patients with MSA-C. The mean age of disease onset was 57.7 ± 8.7 years. The mean disease duration of all patients was 2.9 ± 1.6 years. The frequency of CHS in all patients with MSA was 20.1%, whereas patients with MSA-P had a higher frequency of CHS than patients with MSA-C (25.3 vs. 15.4%; *p* = 0.006).

**TABLE 1:** Demographic and clinical features of the MSA patients with and without CHS.

Variables	All patients				MSA-C			MSA-P					
	All	With CHS	Without CHS	*p* ^all^	All	With CHS	Without CHS	All	With CHS	Without CHS	*p* ^c^	*p* ^p^	*p* ^s^
Number	483	97 (20.1%)	386 (79.9%)	–	254	39 (15.4%)	215 (84.6%)	229	58 (25.3%)	171 (74.7%)	–	–	
Diagnosis (MSA-P/MSA-C)	229/254	58/39	171/215	0.0068	–	–	–		–	–	–	–	
Sex (male/female)	263/220	46/51	217/169	0.120	141/113	20/19	121/94	122/107	26/32	96/75	0.563	0.136	0.898
Age	60.5 ± 8.7	59.9 ± 8.3	60.6 ± 8.8	0.500	59.3 ± 61.7	59.0 ± 7.7	59.4 ± 8.2	61.7 ± 9.2	60.5 ± 8.7	62.1 ± 9.3	0.793	0.261	0.003
Age of onset	57.7 ± 8.7	56.6 ± 8.7	58.0 ± 8.7	0.160	56.9 ± 8.2	56.0 ± 8.3	57.1 ± 8.2	58.6 ± 9.2	57.0 ± 8.9	59.2 ± 9.3	0.450	0.126	0.033
Disease duration	2.9 ± 1.7	3.4 ± 2.0	2.7 ± 1.6	<0.001*	2.6 ± 1.6	3.2 ± 2.0	2.5 ± 1.5	3.2 ± 1.8	3.6 ± 2.0	3.0 ± 0.7	0.011*	0.034	<0.001*
Onset symptoms (Autonomic/Motor)	169/314	46/51	123/263	0.004*	100/154	23/16	77/138	69/160	23/35	46/125	0.006*	0.067	0.034
UMSARS-I	17.0 ± 6.7	20.9 ± 7.8	16.0 ± 6.0	<0.001*	16.6 ± 6.5	22.1 ± 7.9	15.5 ± 5.7	17.5 ± 6.8	20.1 ± 7.7	16.6 ± 6.3	<0.001*	0.001*	0.115
UMSARS-II	18.9 ± 7.1	22.2 ± 8.5	18.1 ± 6.4	<0.001*	17.8 ± 6.7	22.2 ± 8.1	17.0 ± 6.1	20.2 ± 7.3	22.1 ± 8.8	19.5 ± 6.5	<0.001*	0.016	<0.001*
UMSARS-IV	2.2 ± 1.0	2.5 ± 1.2	2.1 ± 0.9	0.001*	2.2 ± 1.0	2.5 ± 1.3	2.1 ± 0.9	2.3 ± 1.0	2.5 ± 1.2	2.2 ± 0.9	<0.001*	0.068	0.063
Total UMSARS score	38.1 ± 13.7	45.6 ± 16.7	36.2 ± 12.2	<0.001*	36.4 ± 13.2	46.9 ± 16.3	34.5 ± 11.6	39.9 ± 14.1	44.7 ± 17.0	38.3 ± 12.7	<0.001*	0.003*	0.005*
OH (yes/no)	232/251	55/177	42/209	0.056	142/112	30/9	112/103	90/139	25/33	65/106	0.004*	0.493	<0.001*
RBD (yes/no)	322/161	67/30	255/131	0.574	170/84	25/14	145/70	152/77	42/16	110/61	0.683	0.260	0.579
Total MOCA score	21.1 ± 5.1	14.5 ± 3.2	14.3 ± 2.8	0.510	21.0 ± 5.4	20.4 ± 5.6	21.1 ± 5.3	21.3 ± 4.9	21.1 ± 4.9	21.3 ± 4.9	0.480	0.729	0.571

*MSA, Multiple System Atrophy; CHS, Cold Hand Sign; UMSARS, Unified Multiple System Atrophy Rating Scale; OH, Orthostatic hypotension; FAB, frontal assessment battery; MoCA, Montreal cognitive assessment; p^c^, comparison between MSA-C patients with CHS and MSA-C patients without CHS; p^p^, comparison between MSA-P patients with CHS and MSA-P patients without CHS; p^s^, comparison between MSA-C patients and MSA-P patients.*

*After Multiple comparison of the p-values adjusted using the false discovery rate approach, which were not significant.

Among all patients with MSA, the patients with CHS had higher UMSARS-I, UMSARS-II, UMSARS-IV, and total UMSARS scores than those without CHS (Table 1). Furthermore, the total NMSS score was higher in patients with CHS than in patients without CHS (Table 2), and most domains showed higher scores in patients with CHS (Table 2), except for domain 5 (attention/memory) and domain 8 (sexual function).

**TABLE 2:** Comparisons of non-motor symptoms between MSA patients with and without cold hand sign.

Variables	All patients				MSA-C			MSA-P					
	All	With CHS	Without CHS	*p* ^all^	All	With CHS	Without CHS	All	With CHS	Without CHS	*p* ^c^	*p* ^p^	*p* ^s^
NMSS total score	67.7 ± 38.5	85.6 ± 42.3	63.2 ± 36.1	<0.001*	64.4 ± 36.1	89.1 ± 41.4	60.0 ± 33.2	71.4 ± 40.8	83.3 ± 43.0	67.3 ± 39.3	<0.001*	0.009*	0.021*
Domain 1 (Cardiovascular including fall)	4.4 ± 5.1	6.6 ± 5.8	3.8 ± 4.7	<0.001*	4.91 ± 5.1	7.6 ± 5.6	4.4 ± 4.9	3.8 ± 5.0	5.8 ± 5.8	3.1 ± 4.4	<0.001*	<0.001*	0.018*
Domain 2 (Sleep/fatigue)	6.4 ± 6.6	8.5 ± 7.4	5.8 ± 6.3	<0.001*	5.7 ± 6.5	8.5 ± 7.8	5.2 ± 6.1	7.1 ± 6.7	8.5 ± 7.1	6.6 ± 6.5	0.003*	0.053	0.029*
Domain 3 (Mood/apathy)	15.6 ± 17.0	19.5 ± 19.2	14.6 ± 16.2	0.011*	14.3 ± 15.7	18.6 ± 18.2	13.5 ± 15.1	17.0 ± 18.2	20.1 ± 20.0	16.0 ± 17.5	0.060	0.137	0.079
Domain 4 (Perceptual problem/hallucination)	0.3 ± 1.5	0.7 ± 2.0	0.2 ± 1.3	0.012*	0.3 ± 1.0	0.6 ± 1.8	0.2 ± 0.8	0.4 ± 1.9	0.7 ± 2.2	0.3 ± 1.8	0.017*	0.170	0.222
Domain 5 (Attention/memory)	4.8 ± 5.2	5.4 ± 5.4	4.7 ± 5.1	0.200	4.6 ± 5.1	6.0 ± 6.2	4.3 ± 4.8	5.1 ± 5.3	5.1 ± 4.9	5.2 ± 5.5	0.063	0.947	0.220
Domain 6 (Gastrointestinal tract)	6.7 ± 6.3	9.6 ± 7.5	6.0 ± 5.7	<0.001*	5.7 ± 5.8	9.7 ± 7.5	5.0 ± 5.1	7.7 ± 6.6	9.4 ± 7.5	7.1 ± 6.2	<0.001*	0.022	0.001*
Domain 7 (Urinary)	19.1 ± 12.2	23.3 ± 10.9	18.1 ± 12.3	<0.001*	18.9 ± 12.4	25.5 ± 10.0	17.8 ± 12.4	19.3 ± 11.9	21.8 ± 11.4	18.5 ± 12.1	<0.001*	0.065	0.743
Domain 8 (Sexual function)	6.3 ± 7.9	**6.9** ± 8.3	6.1 ± 7.8	0.431	6.9 ± 8.2	7.7 ± 9.2	6.7 ± 8.0	5.6 ± 7.4	6.3 ± 7.8	5.4 ± 7.3	0.496	0.440	0.074
Domain 9 (Miscellaneous)	4.2 ± 4.6	5.3 ± 4.5	3.9 ± 4.5	0.007*	3.1 ± 4.1	4.9 ± 4.1	2.8 ± 4.0	5.3 ± 4.8	5.6 ± 4.9	5.2 ± 4.8	0.004*	0.664	<0.001*
HDRS score	12.0 ± 7.9	13.7 ± 9.1	11.6 ± 7.5	0.023*	11.1 ± 7.3	13.3 ± 9.7	10.7 ± 6.7	13.0 ± 8.4	13.9 ± 8.7	12.7 ± 8.3	0.045	0.354	0.008*
HARS score	10.3 ± 7.2	11.6 ± 8.0	10.0 ± 7.0	0.045	9.9 ± 6.7	11.3 ± 7.4	9.6 ± 6.6	10.8 ± 7.8	11.9 ± 8.4	10.5 ± 7.5	0.155	0.232	0.155

*MSA, Multiple System Atrophy; CHS, Cold Hand Sign; NMSS, Non-Motor Symptom Scale; p^c^, comparison between MSA-C patients with CHS and MSA-C patients without CHS; p^p^, comparison between MSA-P patients with CHS and MSA-P patients without CHS; p^s^, comparison between MSA-C patients and MSA-P patients. HDRS, Hamilton depression rating scale; HARS, Hamilton anxiety rating scale.*

*After Multiple comparison of the p-values adjusted using the false discovery rate approach, which were not significant.

In the MSA-C subgroup, patients with CHS showed higher UMSARS-I, UMSARS-II, UMSARS-IV, and total UMSARS scores than those without CHS (Table 1). MSA-C patients with CHS had a higher prevalence of OH than those without CHS (Table 1). In addition, among the MSA-C patients, the total NMSS score was higher in patients with CHS (Table 2), and most domains showed higher scores in patients with CHS except for domain 3 (mood/apathy), domain 5 (attention/memory), and domain 8 (sexual function) (Table 2).

In the MSA-P subgroup, patients with CHS had higher UMSARS-I and total UMSARS scores than those without CHS (Table 1). In addition, the total NMSS score was higher in MSA-P patients with CHS than in those without CHS (Table 2), whereas only the domain 6 (gastrointestinal tract) score was higher in patients with CHS than in those patients without CHS.

In the comparison between MSA-C and MSA-P, patients with MSA-P had a longer disease duration, lower frequency of CHS, higher UMSARS-I, and total UMSARS scores. Moreover, HARS score, total NMSS score, domain 1 (cardiovascular including fall), domain 2 (sleep/fatigue), domain 5 (attention/memory), and domain 9 (miscellaneous) scores were higher in patients with MSA-P than in patients with MSA-C (Table 2).

### Univariate and Multivariate Logistic Regression in the Multiple System Atrophy Cohort

Factors associated with CHS in patients with MSA, MSA-C, and MSA-P in the univariate regression model are shown in Table 3. We found that longer disease duration and higher UMSARS-I, UMSARS-II, UMSARS-IV, and total UMSARS scores were associated with CHS in the MSA, MSA-C, and MSA-P subgroups. In addition, the presence of OH (*p* = 0.006) and UMSARS-IV (*p* < 0.001) were associated with CHS in the MSA-C subgroup, but not in the other two groups. Meanwhile, CHS was associated with a higher NMSS score (*p* < 0.001) in all three MSA group patients, and most domains among NMSS in the MSA and MSA-C subset, but some domains of NMSS in the MSA-P subgroup (Table 3).

**TABLE 3:** Factors associated with CHS in MSA, MSA-P, and MSA-C patients in univariate regression model.

Variables	Total			MSA-C			MSA-P		
	OR	95% CI	*P*-value	OR	95% CI	*P*-value	OR	95% CI	*P*-value
Sex (male/female)	0.702	0.450–1.098	0.121	0.818	0.413–1.619	0.818	0.635	0.349–1.156	0.137
Age	0.991	0.966–1.017	0.499	0.994	0.95–1.037	0.792	0.981	0.950–1.014	0.256
Age of onset	0.982	0.957–1.007	0.160	0.984	0.994–1.026	0.449	0.975	0.944–1.007	0.127
Disease duration	1.246	1.103–1.407	<0.001*	1.262	1.044–1.525	0.016*	1.188	1.011–1.395	0.036
Onset symptoms (Autonomic/Motor)	1.929	1.227–3.032	0.004	2.528	1.245–5.133	0.010*	1.361	0.731–2.533	0.332
UMSARS-I	1.113	1.075–1.153	<0.001*	1.161	1.097–1.228	<0.001*	1.076	1.029–1.126	0.001*
UMSARS-II	1.084	1.050–1.119	<0.001*	1.120	1.063–1.180	<0.001*	1.051	1.009–1.095	0.017
UMSARS-IV	1.449	1.168–1.797	0.001*	1.570	1.139–2.164	0.006*	1.313	0.978–1.763	0.070
Total UMSARS score	1.049	1.032–1.067	<0.001*	1.069	1.041–1.098	<0.001*	1.032	1.010–1.054	0.004*
OH (yes/no)	1.546	0.987–2.422	0.057	3.065	1.389–6.765	0.006*	1.235	0.675–2.261	0.493
RBD (yes/no)	1.383	0.862–2.220	0.179	1.601	0.756–3.387	0.219	1.285	0.690–2.393	0.429
Total MOCA score	0.986	0.944–1.029	0.510	0.978	0.919–1.041	0.479	0.989	0.931–1.051	0.989
HDRS score	1.032	1.004–1.061	0.024*	1.046	1.000–1.094	0.048	1.017	0.983–1.053	0.353
HARS score	1.031	1.000–1.062	0.046	1.036	0.987–1.088	0.156	1.023	0.985–1.063	0.231
NMSS Total score	1.015	1.009–1.020	<0.001*	1.021	1.012–1.031	<0.001*	1.009	1.002–1.017	0.001*
Domain 1 (Cardiovascular including fall)	1.098	1.054–1.143	<0.001*	1.110	1.045–1.179	0.001*	1.107	1.045–1.172	0.001*
Domain 2 (Sleep/fatigue)	1.058	1.025–1.091	<0.001*	1.068	1.020–1.118	0.005*	1.043	0.999–1.088	0.055
Domain 3 (Mood/apathy)	1.016	1.003–1.028	0.012*	1.019	0.999–1.039	0.064	1.012	0.996–1.028	0.139
Domain 4 (Perceptual problem/hallucination)	1.157	1.020–1.313	0.024*	1.311	1.016 = 1.692	0.038	1.099	0.953–1.266	0.194
Domain 5 (Attention/memory)	1.027	0.986–1.070	0.201	1.059	0.996–1.125	0.066	0.998	0.944–1.056	0.946
Domain 6 (Gastrointestinal tract)	1.087	1.051–1.124	<0.001*	1.127	1.066–1.190	<0.001*	1.052	1.007–1.099	0.024
Domain 7 (Urinary)	1.037	1.017–1.057	<0.001*	1.056	1.024–1.089	0.001*	1.024	0.998–1.050	0.066
Domain 8 (Sexual function)	1.011	0.984–1.039	0.430	1.014	0.974–1.056	0.495	1.016	0.977–1.056	0.439
Domain 9 (Miscellaneous)	1.063	1.016–1.113	0.008*	1.109	1.029–1.194	0.007*	1.014	0.953–1.078	0.663

*MSA, Multiple System Atrophy; CHS, Cold Hand Sign; UMSARS, Unified Multiple System Atrophy Rating Scale; OH, Orthostatic hypotension; MoCA, Montreal cognitive assessment; HDRS, Hamilton depression rating scale; HARS, Hamilton anxiety rating scale; NMSS, Non-Motor Symptom Scale.*

*Significant difference after adjusting by false discovery rate.

In the multivariable regression model, the total UMSARS score (OR = 1.009; 95% CI = 1.002–1.017; *p* < 0.001) and NMSS score (OR = 1.009; 95% CI = 1.002–1.017; *p* < 0.001) were associated with CHS in all patients with MSA. Similarly, in the MSA-C group, the total UMSARS score (OR = 1.053; 95% CI = 1.015–1.093; *p* = 0.006) and NMSS scores (OR = 1.015; 95% CI = 1.002–1.017; *p* = 0.025) were also associated with CHS. Only the total UMSARS score (OR = 1.032; 95% CI = 1.010–1.054; *p* = 0.004) was associated with CHS in patients with MSA-P (Table 4).

**TABLE 4:** Factors associated with CHS in MSA, MSA-P, and MSA-C patients in multivariable regression model.

Variables	Total			MSA-C			MSA-P		
	OR	95% CI	*P*-value	OR	95% CI	*P*-value	OR	95% CI	*P*-value
UMSARS score	1.031	1.010–1.053	0.003	1.053	1.015–1.093	0.006	1.032	1.010–1.054	0.004
NMSS score	1.008	1.000–1.015	0.036	1.015	1.002–1.029	0.025	–	–	–

*MSA, Multiple System Atrophy; CHS, Cold Hand Sign; UMSARS, Unified Multiple System Atrophy Rating Scale; NMSS, Non-Motor Symptom Scale.*

### Survival Analysis in Patients With Multiple System Atrophy at the Disease Duration ≤ 3 Years

**FIGURE 2:**
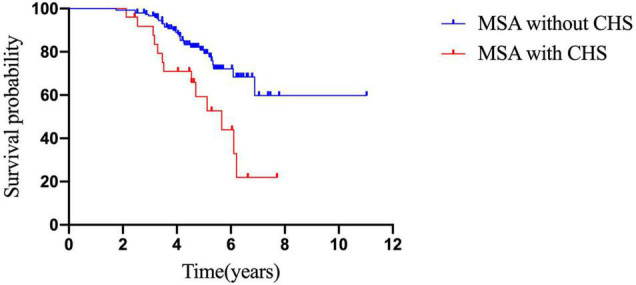
Comparison of survival between patients with and without CHS.

Kaplan–Meier analysis revealed that patients with CHS had a shorter survival time than those without CHS (*p* = 0.04; Figure 2). In addition, Cox survival analysis showed that CHS was associated with shorter survival after adjusting for baseline diagnosis subtype, age, sex, OH, disease duration, autonomic onset, total UMSARS, and NMSS scores (*p* = 0.001; HR = 3.701; 95% CI = 1.765–7.760; Table 5).

**TABLE 5:** Cox survival analysis results in MSA patients at the disease duration ≤ 3 years.

Variables	HR	95% CI	*P* *
CHS	3.701	1.765–7.760	0.001

*MSA, Multiple System Atrophy; CHS, Cold Hand Sign; OH, Orthostatic hypotension UMSARS, Unified Multiple System Atrophy Rating Scale, NMSS, Non-Motor Symptom Scale.*

*After adjusting for baseline diagnosis subtype, age, sex, OH, disease duration, autonomic onset, score of total UMSARS and score of NMSS.

## Discussion

This study was the first extensive research that focused on the frequency and correlative factors of CHS in MSA and investigated the early occurrence of CHS for the survival of MSA. Our study found that CHS was not rare in MSA, and motor and non-motor symptoms were associated with CHS, not only in the entire MSA group but also in the MSA subtypes. In addition, we also found that early occurrence of CHS in patients with MSA was associated with a shorter survival time, indicating a poorer prognosis.

This study focused on the frequency of CHS and correlative factors of CHS in MSA, which was one of the “red flags” in the diagnosis of MSA (Kollensperger et al., 2008). We found that the frequency of CHS in all patients with MSA was 20.1%, which was similar to an Indian study that reported that 20% of patients with MSA presented with CHS (Tandon and Pradhan, 2015). However, the frequency of CHS among our MSA-P cohort (25.3%) was lower than that from another Caucasian study (including 57 patients with MSA-P), showing that 39% of these patients had CHS (Kollensperger et al., 2008); these findings collectively indicate that CHS in MSA-P was not rare and patients with MSA-P complaining of CHS were more common than patients with MSA-C. For the differential frequency of CHS among different studies, several reasons should be considered. First, the ethnic and genetic backgrounds, education, and cognition of CHS were different. Second, the composition of patients may be another reason for differential frequencies, such as the ratio of subtypes (MSA-C/MSA-P). As shown in our study, the frequency of CHS in MSA-P was higher than that in MSA-C (25.3 vs. 15.4%). A previous study also found that the frequency of CHS was higher in patients with MSA-P than in patients with MSA-C, as well as some other dysautonomic symptoms (Schmidt et al., 2008). The different patterns of CHS in MSA-C and MSA-P may reflect distinct pathological changes in the different subtypes. For example, previous studies have shown that in addition to glial cell α-synuclein inclusions found in both MSA-C and MSA-P subgroups, MSA-P is more vulnerable to striatonigral degeneration, whereas MSA-C is more likely to be affected by olivopontocerebellar atrophy (Hashimoto et al., 2008; Ahmed et al., 2012; Jellinger, 2014). Third, we found that disease duration plays a role in the occurrence of CHS in MSA. On the one hand, the disease duration in our patients (2.9 ± 1.7 years) was shorter than that in the previous European study (4.9 ± 3.8 years), and the frequency of CHS was lower than that in the previous study (Kollensperger et al., 2008). On the other hand, in our study, the frequency of CHS was 8.2% in patients with disease duration < 5 years, while it increased to 33.9% in the subgroup with disease duration > 5 years. However, in the multivariable regression analysis, we found that only the total UMSARS and NMSS scores were associated with CHS, but the disease duration was not associated with CHS, which suggested that disease severity was an independent factor for CHS, while disease duration was not. These results suggest that disease severity plays a core role in the occurrence and development of CHS.

In our study, we found that disease severity measured using UMSARS and NMSS was related to CHS in patients with MSA. Although a previous study found that there was no difference in disease duration and disease severity (Hoehn and Yahr staging score) between MSA with CHS (*n* = 18) and MSA without CHS patients (*n* = 6), there were trends that patients with CHS had longer disease duration (6.8 ± 1.9 vs. 5.2 ± 2.7 years) and severer Hoehn and Yahr staging (4.3 ± 1.0 vs. 3.7 ± 1.1) than that in MSA without CHS (Pietzarka et al., 2010). The small sample size and the absence of a specific scale such as UMSARS to assess disease severity might contribute to this difference between this study and ours. However, our study found that CHS was not correlated to cognition, depression, and anxiety in the regression model, suggesting that CHS was an independent feature of MSA.

Contrarily, we found that CHS was an independent risk factor associated with shorter survival, which was not affected by age at onset, sex, disease subtype, OH, disease duration, autonomic onset, total UMSARS, and NMSS score. Previous studies have shown that severe dysautonomia, early occurrence of autonomic failure, and OH predicted the poor prognosis of MSA (Coon et al., 2015; Low et al., 2015; Cao et al., 2018), which supports our findings because CHS is caused by disturbed neurovascular thermoregulation of distal extremities and also belongs to autonomic features of MSA. Furthermore, pathophysiological studies have indicated that cutaneous sympathetic vasoconstrictor activity is compromised in patients with MSA, which might be attributable to the skin vasomotor center in the medulla oblongata and hypothalamus (Mano et al., 2006; Shindo et al., 2020). In addition, prolonged vasoconstriction of the cutaneous vessels could cause CHS in patients with MSA and impaired peripheral autonomic function (Shindo et al., 2017). Therefore, both pre- and postganglionic skin vasomotor dysfunction may contribute to CHS related to the impaired peripheral circulatory pathway (Mano et al., 2006; Shindo et al., 2017, 2020).

## Conclusion

Our study demonstrated that CHS was common in patients with MSA, especially MSA-P. Greater disease severity and severe non-motor symptoms were associated with CHS in patients with MSA. Similar to other autonomic symptoms, patients with early CHS have a poor prognosis.

## Data Availability Statement

The datasets presented in this article are not readily available because of patient privacy reasons. Requests to access the datasets should be directed to HS, hfshang2002@126.com.

## Ethics Statement

The studies involving human participants were reviewed and approved by the Ethics Committee of West China Hospital of Sichuan University. The patients/participants provided their written informed consent to participate in this study.

## Author Contributions

BC: (1) research project: A. conception, B. organization, C. execution, (2) statistical analysis, design, (3) manuscript: writing of the first draft. YL: (1) research project: A. conception, B. organization, C. execution, (2) statistical analysis, design, (3) manuscript: writing of the first draft. L-YZ, Y-BH, R-WO, and Q-QW: patients enrollment and follow up. HS: (1) research project: conception, (2) statistical analysis: review and critique, (3) manuscript: review and critique. All authors contributed to the article and approved the submitted version.

## Conflict of Interest

The authors declare that the research was conducted in the absence of any commercial or financial relationships that could be construed as a potential conflict of interest.

## Publisher’s Note

All claims expressed in this article are solely those of the authors and do not necessarily represent those of their affiliated organizations, or those of the publisher, the editors and the reviewers. Any product that may be evaluated in this article, or claim that may be made by its manufacturer, is not guaranteed or endorsed by the publisher.
